# Polo-Like Kinase-1 Controls Aurora A Destruction by Activating APC/C-Cdh1

**DOI:** 10.1371/journal.pone.0005282

**Published:** 2009-04-23

**Authors:** Renske van Leuken, Linda Clijsters, Wouter van Zon, Dan Lim, XueBiao Yao, Rob M. F. Wolthuis, Michael B. Yaffe, René H. Medema, Marcel A. T. M. van Vugt

**Affiliations:** 1 Department of Medical Oncology, University Medical Centre Utrecht, Utrecht, the Netherlands; 2 Division of Molecular Biology, The Netherlands Cancer Institute, Amsterdam, the Netherlands; 3 The David H. Koch Institute for Integrative Cancer Research at Massachusetts Institute of Technology, Cambridge, Massachusetts, United States of America; 4 University of Science and Technology, Hefei, China; 5 Department of Medical Oncology, University Medical Center Groningen, Groningen, The Netherlands; Dartmouth College, United States of America

## Abstract

Polo-like kinase-1 (Plk1) is activated before mitosis by Aurora A and its cofactor Bora. In mitosis, Bora is degraded in a manner dependent on Plk1 kinase activity and the E3 ubiquitin ligase SCF-βTrCP. Here, we show that Plk1 is also required for the timely destruction of its activator Aurora A in late anaphase. It has been shown that Aurora A destruction is controlled by the auxiliary subunit Cdh1 of the Anaphase-Promoting Complex/Cyclosome (APC/C). Remarkably, we found that Plk1-depletion prevented the efficient dephosphorylation of Cdh1 during mitotic exit. Plk1 mediated its effect on Cdh1, at least in part, through direct phosphorylation of the human phosphatase Cdc14A, controlling the phosphorylation state of Cdh1. We conclude that Plk1 facilitates efficient Aurora A degradation through APC/C-Cdh1 activation after mitosis, with a potential role for hCdc14A.

## Introduction

The transition from G2 to mitosis is triggered by rapid activation of the Cyclin B1/Cdk1-complex [1]. Polo-like kinase 1 (Plk1) positively influences mitotic entry by activating the Cdk1-activating Cdc25 phosphatases and by inducing the ubiquitin-dependent destruction of the Cdk1-repressor Wee1 [2], [3]. Plk1 phosphorylation initiates the destabilization of Wee1 by creating a recognition sequence for the F-box protein β-TrCP that cooperates with the SCF ubiquitin-ligase [4].

In late G2, Plk1 is activated by a pathway depending on Bora and Aurora A, resulting in phosphorylation of Threonine 210 (T210) in its activating T-loop [5]. Plk1 activation is particularly important when cells need to recover from a DNA damage-dependent G2 arrest [6]. In addition to targeting Wee1 for destruction, re-activation of Plk1 reinitiates the cell cycle and promotes mitotic entry by destabilizing Claspin, an adaptor protein required for Chk1-activation [7]–[9]. Plk1 further controls the β-TrCP-dependent destruction of the APC/C-inhibitor Emi-1 and the mitotic regulator Bora [10]–[14]. Altogether, Plk1 exerts many of its effects on the G2/M transition by promoting the timely destruction of critical cell cycle regulators.

Further progression through mitosis requires the timely degradation of mitotic regulators by the Anaphase-Promoting Complex or Cyclosome (APC/C). The APC/C acts together with one of the WD40 co-activators Cdc20 (homologous to Drosophila Fizzy, *S.pombe* Slp1) or Cdh1 (Cdh1 or Hct1 in *S. cerevisiae*, Fizzy-related in *D. Melanogaster* and Srw1/Ste9 in *S. pombe*)) [15]–[18], (reviewed in [19]). In prometaphase, APC/C-Cdc20 directs the degradation of Nek2a and Cyclin A in a manner dependent on mitotic APC/C phosphorylation [20], [21]. In metaphase, APC/C-Cdc20 targets Cyclin B1 and Securin as soon as the spindle assembly checkpoint is satisfied [22]–[24]. The Cdh1-dependent APC/C is kept inactive in mitosis through direct phosphorylation of Cdh1 by Cyclin B1-Cdk1, which prevents premature mitotic exit [25], [26]. Upon Cyclin B1 destruction and complete Cdk1 inactivation at some point in late anaphase, APC/C-Cdh1 is activated, resulting in the destruction of Cdc20, Plk1, and particularly Aurora A [23], [27]–[32]. Timely activation of APC/C-Cdh1 thus requires release of inhibitory Cdh1 phosphorylation by phosphatases, such as the Cdc14 phosphatase in budding yeast, which controls the association of Cdh1 to the APC/C and renders APC/C-Cdh1 fully active [23], [33].

It has been demonstrated that APC/C activity is regulated through phosphorylation by mitotic kinases. In budding yeast, both mitotic Cyclin-Cdk complexes as well as the *Polo*-related Cdc5 kinase were shown to be required for APC/C activity [27], [32]. In line with these observations, multiple APC/C subunits are phosphorylated by mitotic Cyclin-Cdk and Polo-like kinases *in vitro* and *in vivo* [34]–[36]. However, despite the observed phosphorylation of the APC/C by Plk1, a clear defect in APC/C activation was not observed in Plk1-depleted cells [35], [37], [38]. This might be due to the fact that previously only APC/C-Cdc20 targets were studied in detail after Plk1-depletion. Here, we investigated the role of Plk1 in the activation of both APC/C-Cdc20 and APC/C-Cdh1.

## Results

**Figure 1:**
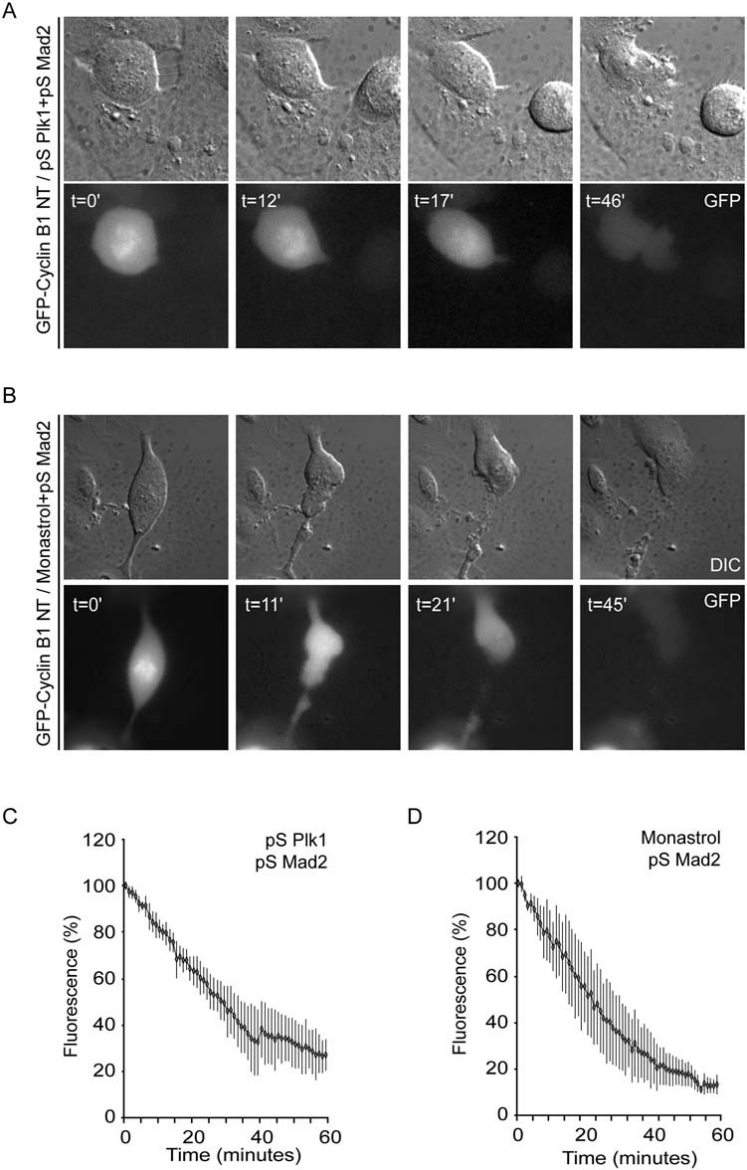
APC/C-Cdc20 Activity in Plk1-depleted U2OS cells. A–B U2OS cells were transiently transfected with 1 µg of GFP-Cyclin B1-NT, 10 µg of pS-Plk1 and 10 µg of pS-Mad2 as indicated. 18 h after transfection, cells were incubated for 24 h in thymidine. 10 h after washing away thymidine, cells were transferred to the heated stage of a time-lapse microscope. At indicated time points, DIC images and fluorescent light were analyzed. Directly after washing away thymidine, monastrol was added to culture medium, where indicated. C, D Fluorescence levels were quantified using Metamorph software (n = 5 for each condition). Quantified images were plotted from the time of mitotic entry as define by nuclear envelope breakdown (t = 0) and the standard error of the mean of 5 experiments is indicated.

To determine APC/C activation in Plk1-depleted cells, we first followed the destruction of a GFP-tagged N-terminal fragment of Cyclin B1 (comprising its destruction-box, but lacking the ability to interact with Cdk1, further referred to as GFP-Cyclin B1-NT) [39] (Suppl. Fig. S1A). In control cells, degradation of GFP-Cyclin B1-NT initiated as soon as chromosomes aligned in control U2OS cells (Suppl. Fig. S1A, B). Anaphase often started before GFP-Cyclin B1-NT was completely degraded, which reflects the inability of this Cyclin B1 fragment to inhibit anaphase onset. Disruption of the destruction box in this GFP-Cyclin B1-NT fragment (GFP-Cyclin B1-NT-DM) rendered it stable during mitosis, and did not interfere with chromosomal localization of GFP-Cyclin B1-NT nor mitotic progression (Suppl. Fig. S1C, D). When we subsequently analyzed GFP-Cyclin B1-NT in Plk1-depleted cells, GFP-Cyclin B1-NT fluorescence remained high because Plk1-depleted cells almost invariably entered mitosis with monopolar or otherwise abnormal spindles, and consequently arrested in pro-metaphase due to the action of the spindle assembly checkpoint, precluding normal degradation of Cyclin B1 [38]. In order to follow APC/C activity in Plk1-depleted cells, we therefore silenced spindle-assembly checkpoint function through simultaneous interference with expression of Mad2. Next, we analyzed Cyclin B1 destruction in mitosis (Fig. 1A). Interestingly, Plk1/Mad2-depleted cells efficiently degraded GFP-Cyclin B1-NT (Fig. 1A), with kinetics very similar to Monastrol-treated control cells (Fig. 1) confirming that Plk1 is not required for activation of spindle checkpoint-dependent APC/C-Cdc20 activity. As a comparison, we also analyzed Cyclin B1 degradation in mitotic cells with monopolar spindles that do express Plk1. To accomplish this, Mad2-depleted cells were treated with monastrol, an inhibitor of Eg5 that blocks centrosome separation but does not alter Plk1 activity [38], [40]. Very similar kinetics of GFP-Cyclin B1-NT degradation were observed in monastrol-treated cells (Fig. 1B and 1D) and Plk1-depleted cells, which shows that Plk1 does not influence APC/C-Cdc20 activity in checkpoint-compromised cells (Fig. 1).

**Figure 2:**
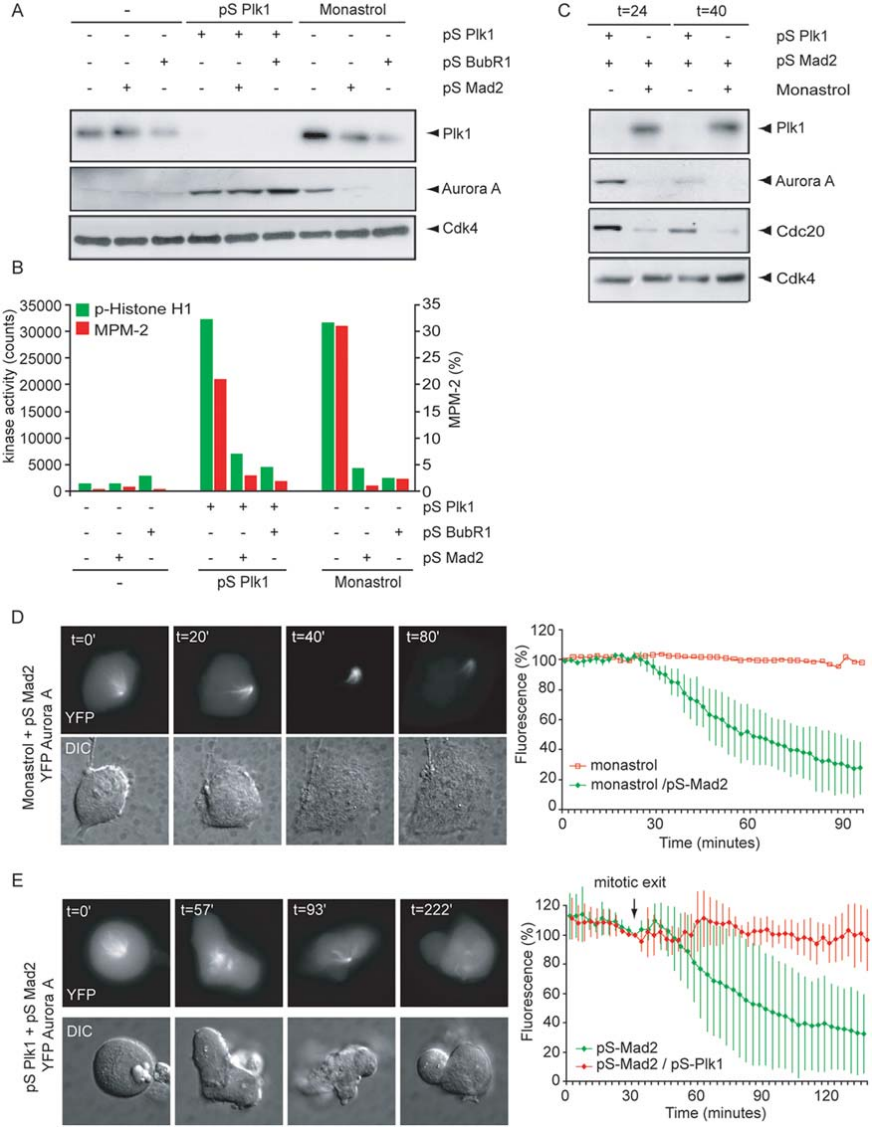
APC/C-Cdh1 activity in Plk1-depleted cells. A, B U2OS cells were transfected with 1 µg pBabePuro, 10 µg pS-Plk1, 10 µg pS-BubR1 or pS-Mad2 as indicated. 18 h after transfection, cells were incubated in thymidine and puromycin for 24 h. After washing away thymidine and puromycin, cells were treated with monastrol if indicated. Cells were harvested at 18 h after release from the thymidine block. Western blotting of whole cell lysates was performed with anti-Plk1, anti-Aurora A and anti-Cdk4. B Alternatively, cells were fixed in ethanol, stained with anti-MPM-2 and analyzed by FACS. MPM2-positivity of 1*10^4^ events is plotted. In parallel, Cyclin B1-associated kinase was measured towards Histone H1. C U2OS cells were treated as for Figure 2A and harvested at 24 h or 40 h after release from a thymidine bock. Western blotting was performed with anti-Plk1, anti-Aurora A and Cdc20 and anti-Cdk4. D, E U2OS cells were transiently transfected with 1 µg Aurora A-YFP, 10 µg pS-Mad2 and 10 µg pS-Plk1 if indicated. 18 h after transfection, cells were incubated for 24 h in thymidine. Directly after washing away thymidine, monastrol was added to the culture medium if indicated. 10 h after washing away thymidine, cells were transferred to the heated stage of a time-lapse microscope. At indicated time points, DIC and fluorescent images were recorded. Fluorescence levels were quantified using Metamorph software (n = 7 for each condition) and arbitrarily set at 100% at mitotic exit (t = 0). Vertical lines indicate the standard error of the mean of 7 experiments.

During the later stages of mitosis, Cdh1 replaces Cdc20 on the APC/C to form the APC/C-Cdh1 complex [23], [25], [26], [33]. APC/C-Cdh1 has a broader substrate specificity when compared to APC/C-Cdc20 and can conjugate ubiquitin to proteins containing a D-box or a KEN-box including Plk1, Aurora A, Cdc20 and Cdc6 [23], [27], [29]–[32], [41]. To study degradation patterns of APC/C-Cdh1 substrates, we analyzed two mitotic regulators, of which the destruction depends on APC/C-Cdh1-activity; Aurora A and Cdc20 [28], [30], [31]. At 18 h after thymidine wash-out, when the majority of cells had exited mitosis, Aurora A levels had almost completely disappeared (Fig. 2A and data not shown). At this time residual levels of Plk1 could be detected, consistent with the finding that Plk1 is only partially degraded during mitotic exit [29]. Cells depleted of Plk1 as well as monastrol-treated cells showed increased Aurora A levels, as was expected from spindle assembly checkpoint-arrested cells (Fig. 2A). Depletion of Mad2 or BubR1 allowed these cells to exit from mitosis, as judged by a drop in MPM2-positivity and a decrease in Cyclin B1-associated kinase activity (Fig. 2B). However, whereas spindle assembly checkpoint silencing led to efficient degradation of Aurora A in monastrol-treated cells, it did not promote Aurora A degradation in Plk1-depleted cells (Fig. 2A). These effects persisted when checkpoint-silenced cells were analyzed at 24 h and 40 h after release from a thymidine block (Fig. 2C). Accordingly, also the degradation of Cdc20 was impaired in Plk1-depleted cells (Fig. 2C), indicating that Plk1 is required for general APC/C-Cdh1 activation.

To analyze the effects of Plk1-depletion on APC/C-Cdh1 activation in more detail, we analyzed YFP-tagged Aurora A using time-lapse microscopy. We confirmed that in control cells Aurora A-YFP levels decreased during mitotic exit [29] (Suppl. Fig. S1E). In contrast to spindle assembly checkpoint-proficient cells, monastrol treatment of spindle assembly checkpoint-deficient cells did not interfere with the degradation of Aurora A-YFP during mitotic exit (Fig. 2D), indicating that the presence of a bipolar spindle is not essential for APC/C-Cdh1 activation in these cells. Next, we monitored degradation of Aurora A in Plk1-depleted cells, in particular in those cells that formed a monopolar spindle, to ensure that we were analyzing cells in which Plk1 was functionally inactivated [38]. Interestingly, fluorescence levels of Aurora A-YFP remained high during mitotic exit in Plk1-depleted cells (Fig. 2E), suggesting that Plk1 is required for degradation of Aurora A by the APC/C-Cdh1.

**Figure 3:**
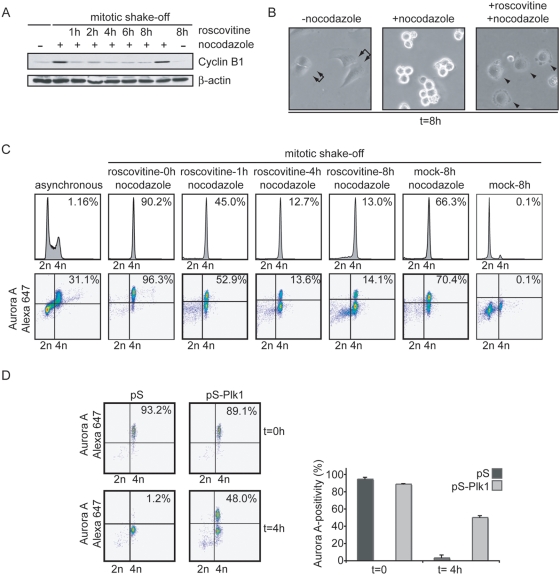
Aurora A degradation during synchronized mitotic exit. A, B, C U2OS cells were treated with nocodazole for 16 hours. Mitotic cells were collected by mitotic shake off and left untreated or treated with Roscovitine. At indicated time points after Roscovitine addition, cell lysates were obtained (A), DIC images were takes (B) or cells were fixed in ethanol for FACS analysis (C). Cell lysates were analyzed by Western blotting using anti-Cyclin B1 and anti-β-Actin (A). In parallel, cells were co-stained with anti-Aurora A-Alexa 674 and anti-MPM-2-Alexa 488 and analyzed by FACS. MPM2-positivity of 1*10^4^ events is indicated in upper-right corner of DNA profile histograms. Aurora A-positivity is indicated in upper right corner of Aurora A plots. D. U2OS cells were transfected with pS or pS-Plk1 in combination with GFP-spectrin. 36 h after transfection, nocodazole was added to cell cultures. After 16 h, mitotic cells were collected by shake-off. Mitotic cells were replated in the absence or presence of Roscovitine for 4 h, and subsequently cell were fixed in ethanol and stained with anti-Aurora A-Alexa-647. Representative Aurora A-plots of GFP-positive cells are shown (left panel) and the mean and SEM of 3 experiments are plotted (right panel).

**Figure 4:**
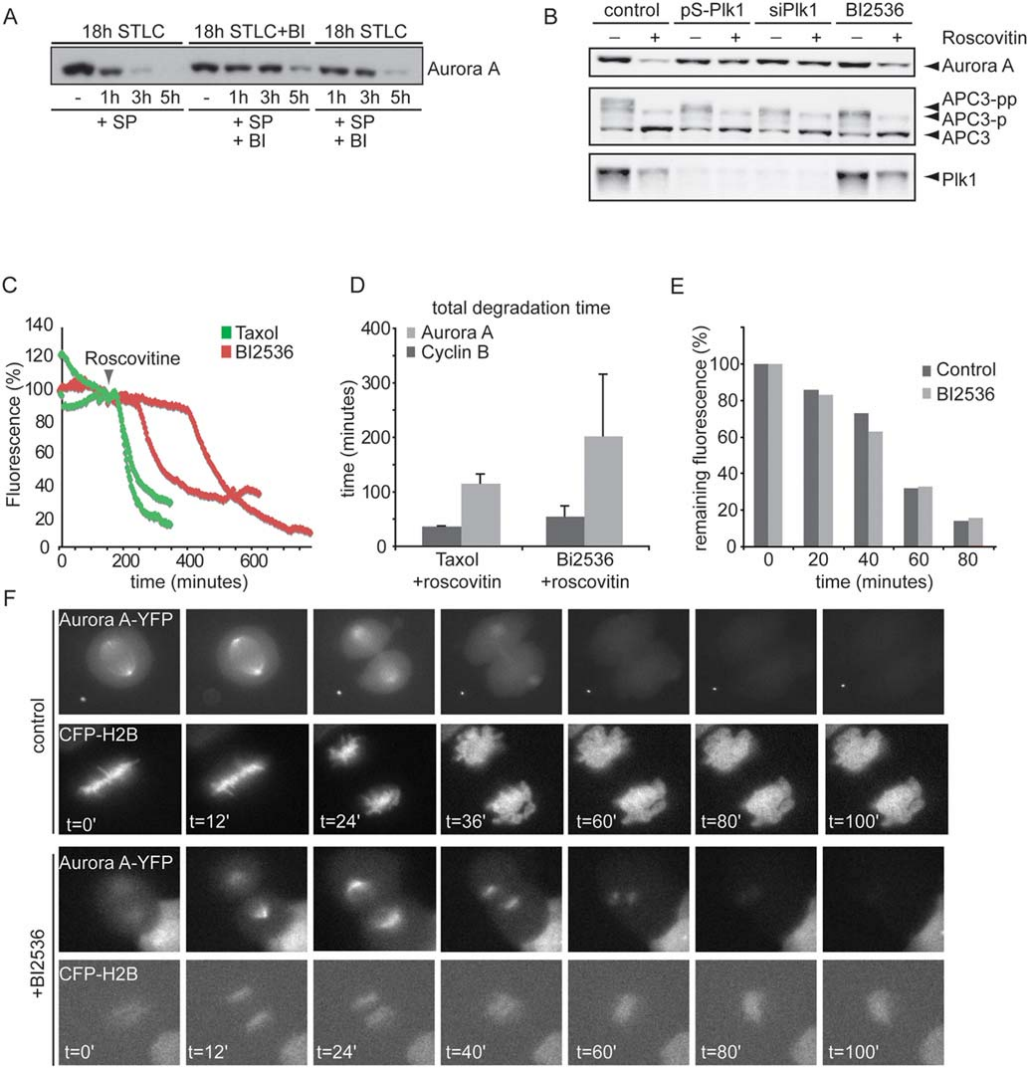
Plk1 activity is required to control Aurora A levels during mitotic exit. A. Cells were synchronized by treatment with 20 µM STLC for 18 h in the presence (BI) or absence of 20 nM BI 2536. Mitotic cells were then harvested by mitotic shake-off. Cells were replated in medium with STLC, BI2536 and immediately induced to exit from mitosis by addition of the Mps1 inhibitor SP600125, either alone (lanes 1–4) or in combination with 20 nM BI 2536 (lanes 5–8). Addition of BI2536 after release of the spindle checkpoint was insufficient to stabilize Aurora A to the same extent (Fig 4A, lane 9–12). At the indicated time-points samples were prepared in sample buffer and analyzed on Western blots with the indicated antibodies. B. U2OS cells were co-transfected with 1 µg pBabepuro vector and either 10 µg pS or pS-Plk1 vectors (and selected) or tranfected with a si-RNAi pool targeting Plk1. Cell were thymidine released and subsequently arrested in mitosis by adding Nocodazole to pS transfected cells, BI2536 to untreated cells, or intrinsically by Plk1-RNAi. Mitotic cells were collected by mitotic shake off. Half of the mitotic cells were replated in the presence of Roscovitin to force cells into G1 (lanes with +), the other half of the mitotic cells were also replated but kept in mitosis (pS mitotic cells with nocodazole, pS-Plk1 mitotic cells intrinsically and untreated cells with BI 2536, lanes with −). Cells were collected after 1 hour, lysed and protein levels were measured. Whole cell extracts were probed for APC3, Aurora A and Plk1. C, D U2OS cells were transfected with 0.1 µg Aurora A-YFP A and 0.1 µg Cyclin B1-Cherry. Following release form a thymidine block cells were incubated with taxol or BI2536. Roscovitin was added to induce mitotic exit and fluorescence images were obtained at indicated timepoints. In Plk1-depleted cells, onset of Aurora A destruction was first delayed and slow when mitotic exit was visible. D. Averages and standard deviations of total degradation times for Cyclin B and Aurora A were plotted (12 cells). E. U2OS cells transfected with Aurora A-YFP A and H2B-CFP were treated with or without BI2536 after chromosome alignment was completed. YFP levels during mitotic progression were measured. F. Graphs show quantification of representative images of YFP fluorescence from metaphase to the next G1 in cells treated with or without BI2536.

Activation of APC/C-Cdh1 could be a late mitotic function of Plk1, as APC/C-Cdh1 is normally not activated prior to metaphase. However, studying late mitotic functions of Plk1 is complicated by the fact that inhibition of Plk1 causes multiple early mitotic defects, most notably spindle defects [38]. To circumvent this issue, we used an experimental setup that allowed synchronized mitotic exit in spindle-checkpoint arrested cells. In normal mitosis Cdh1 is prevented from binding to the APC/C through direct inhibitory phosphorylation by Cyclin B1-Cdk1 [25], [26], but mitotic exit and activation of APC/C-Cdh1 can be initiated by the addition of a Cdk-inhibitor to prometaphase cells [42]. Therefore, nocodazole-treated mitotic cells were collected by mitotic shake-off and replated in the presence of nocodazole and the Cdk1 inhibitor Roscovitine. This resulted in rapid degradation of Cyclin B1 (Fig. 3A) and 4N DNA containing post-mitotic cells (Fig. 3B, C), as cytokinesis fails in the absence of a functional spindle. Clearly, APC/C-Cdh1 was rapidly activated in control cells as judged by degradation of Aurora A (Fig. 3C). In contrast, in Plk1-depleted cells, Aurora A was not degraded (Fig. 3D), whereas these cells did exit mitosis as judged by DNA decondensation and re-attachment to the culture dish (data not shown). Importantly, the impaired degradation of Aurora A after RNA interference of Plk1, was reversed by expression of a RNAi-resistant Plk1, indicating that the observed effects are specific for Plk1 (Suppl. Fig. S2C). Taken together, these data are consistent with a model in which Plk1 is required for Aurora A degradation through activation of the APC/C-Cdh1.

We next wondered when Plk1 kinase activity was required for degradation of Aurora A as a result of APC/C-Cdh1 activation. To resolve this, we made use of a selective inhibitor of Plk1, BI2536, to acutely inactivate Plk1 at different stages in mitosis [43], [44]. For this, cells were first arrested in mitosis with S-trityl-L-cysteine (STLC), an inhibitor of Eg5 that causes monopolar spindles [45]. Subsequently, cells were forced to exit mitosis by addition of SP600125, which inactivates the spindle assembly checkpoint by direct inhibition of the essential checkpoint kinase Mps1 [46]. In the absence of BI2536, addition of SP600125 to STLC-arrested cells results in rapid and efficient degradation of Aurora A (Fig. 4A), indicating that the APC/C-Cdh1 is normally activated in this experimental setting. However, when BI2536 was added to the cells during the STLC-induced prometaphase arrest, well before the cells were forced out of mitosis, Aurora A degradation was delayed (Fig. 4A, lane 5–8), consistent with the effect of Plk1 RNAi on Aurora A degradation, albeit that the overall effect was less dramatic.

To further compare the effect of inhibiting Plk1 kinase activity by BI2536 with depletion of Plk1 by RNAi, we compared both treatments in combination with direct Cdk-inhibition to promote mitotic exit of spindle checkpoint-arrested cultures. To this end, mitotic cells obtained by treatment with monastrol, BI2536, or Plk1 RNAi (short hairpin pS-Plk1 and Plk1 RNAi oligo's) were induced to exit mitosis by the addition of Roscovitine. Consistent with what we found using flow cytometry (Fig. 3D), Plk1 RNAi blocked the degradation of Aurora A (Fig. 4B). Similarly, treatment with BI2536 also inhibited degradation of Aurora-A, but to a lesser extent (Fig. 4B), indicating that apart from Plk1 kinase activity, activation of APC/C-Cdh1 by Plk1 requires a structural contribution from Plk1. A requirement for Plk1 kinase activity for Aurora A degradation was confirmed using time-lapse microscopy (Fig. 4C, D). U2OS cells, expressing Aurora A-YFP A as well as Cyclin B1-Cherry were arrested in the spindle checkpoint through addition of taxol or BI2536 and were subsequently forced to exit mitosis by addition of roscovitin. Whereas taxol-treated cells degraded Aurora A shortly after roscovitin addition, BI2536-treated cells showed delayed Aurora A degradation kinetics, whereas degradation of Cyclin B1-Cherry continued efficiently (Suppl. Fig. S2D and Fig. 4C, D).

To investigate the requirement for Plk1 after the checkpoint is turned off, we again turned to time-lapse microscopy and introduced Aurora A-YFP in combination with CFP-H2B. Now, BI2536 was added when cells had reached full chromosome alignment, as judged from the CFP-H2B images (Fig. 4E, F). Interestingly, addition of BI2536 at metaphase, at a stage when the checkpoint is turned off, failed to inhibit Aurora-A degradation (Fig. 4E, F), indicating that Plk1 is no longer required for APC/C-Cdh1 activation once cells reach metaphase. This could suggest that Plk1-dependent activation of APC/C-Cdh1 occurs prior to metaphase, at a time APC/C-Cdh1 activity is still restrained by Cyclin B1-Cdk1 activity.

The phosphorylation state of Cdh1 was previously shown to determine both Cdh1 association with the APC/C as well as activity of APC/C-Cdh1 [23], [25], [26], [47]. Evidence from *S. cerevisiae* has shown that the Cdc14 phosphatase dephosphorylates Cdh1 to mediate mitotic exit [48]. In order to accomplish Cdh1 dephosphorylation, the Mitotic Exit Network (MEN) is required for the release of Cdc14 from the nucleolus [49]. In human cells however, the upstream regulation of Cdh1 during mitotic exit is less well studied. Interestingly, one of the two human Cdc14 homologues, hCdc14A, can efficiently dephosphorylate Cdh1 and activate APC/C-Cdh1 *in vitro* [47].

**Figure 5:**
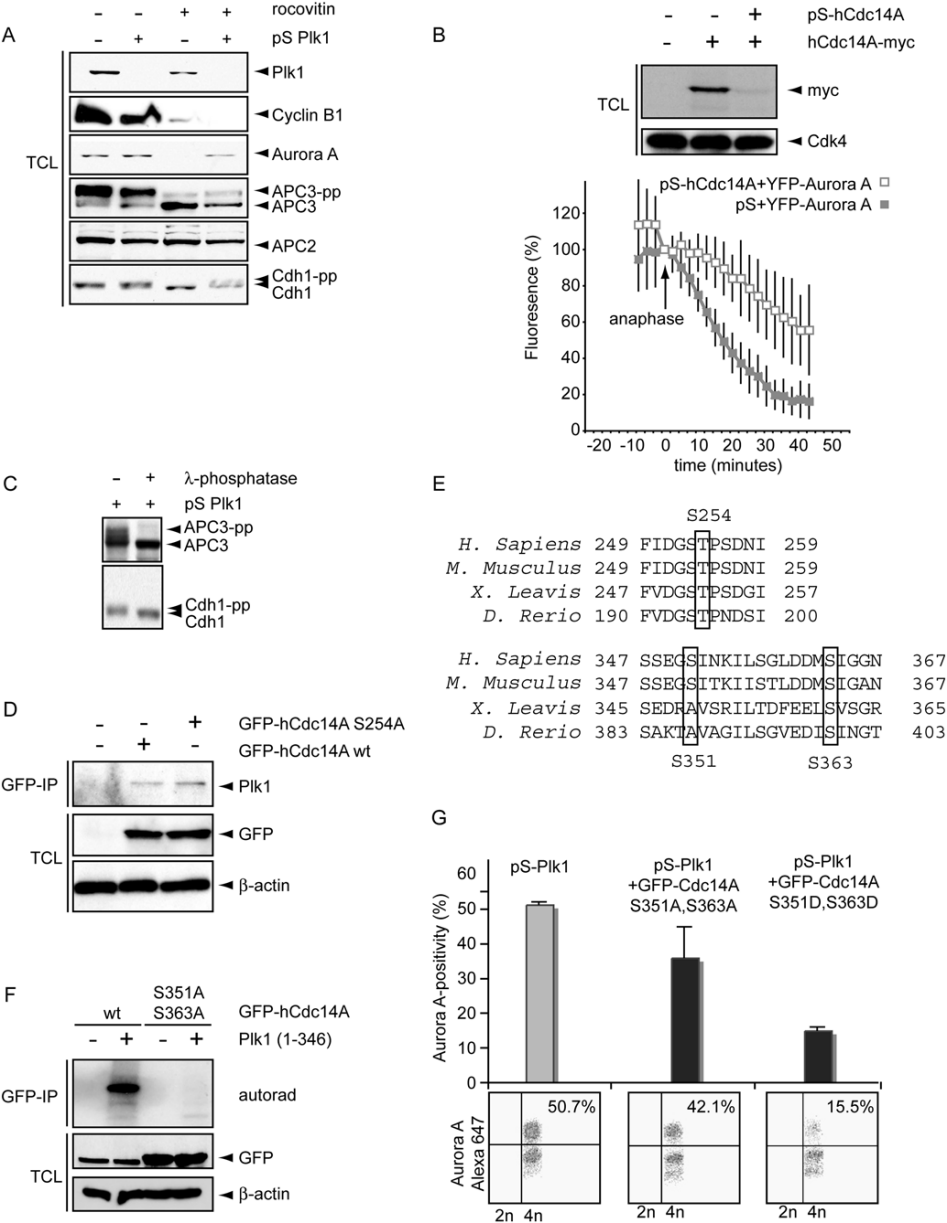
Plk1 controls Cdc14A to regulate the APC/C-Cdh1. A. U2OS cells were treated with pS or pS-Plk1 as in Figure 4B. Cell lysates were obtained after 2 h of Roscovitine treatment. Total cell lysates were blotted for Plk1, Cyclin B, Aurora A, APC3, APC2 and Cdh1. B. U2OS cell were transfected with hCdc14A-myc in combination with pS-hCdc14A. Cell lysates were immunoblotted for Cdk4 and myc. In parallel, U2OS cells were transfected with Aurora A-YFP A in combination with pS or pS-hCdc14A. After release from a thymidine block cells were analyzed using live microscopy. Aurora A levels were quantified and mean and SEM of 5 movies were synchronized at anaphase onset. After anaphase onset, 50% degradation of Aurora A was reached on average after 41 minutes for pS-Cdc14A transfected cells versus 17 minutes in control cells. C. U2OS cells were transfected with pS-Plk1and mitotic cells were collected by mitotic shake-off 60 h after transfection, and subsequently lysed. Extracts were incubated in the presence or absence of lambda-phosphatase. Western blots were probed with anti-APC3 or anti-Cdh1. D. U2OS cells were transfected with wt-GFP-hCdc14a or GFP-hCdc14A-S254A. After 48 h, GFP-immunoprecipitations were performed and total cell lysates and GFP-IP's were immunoblotted for GFP, Plk1 and β-Actin. E. The conservation in the regions comprising Ser254, Ser351 and Ser363 of Cdc14A is indicated. F. U2OS cells were transfected with wt-GFP-hCdc14a or GFP-hCdc14A S351, 363A. After 48 h, GFP-immunoprecipitations were performed. GFP-immunoprecipitations were left untreated or incubated with recombinant Plk1. Kinase reactions were visualized by autorad. Total cell lysates were immunoblotted for GFP and β-Actin (lower panels). G. U2OS cells were transfected with pS-Plk1 in combination with GFP-hCdc14A S351,363A or GFP-hCdc14A S351,363D. 36 h after transfection, nocodazole was added to cell cultures. After 16 h, mitotic cells were collected by shake-off. Mitotic cells were subsequently replated in presence of Roscovitine for 4 h, and subsequently cells were fixed in ethanol and stained with anti-Aurora A-Alexa-647. Representative Aurora A-plots of GFP-positive cells are shown (lower panel) and the mean and SEM of 3 experiments are plotted (upper panel).

To investigate if Cdc14-dependent dephosphorylation of Cdh1 might be regulated by Plk1 in human cells, we analyzed Cdh1 behavior in Plk1-depleted cells forced to exit mitosis by the addition of Roscovitine. As expected, Cyclin B1 and Aurora-A are rapidly degraded in the control cells after treatment with Roscovitine (Fig. 5A). Interestingly, the Cdk1 target APC3 was dephosphorylated after treatment with Roscovitine in control and Plk1-depleted cells (Fig. 5A), indicating that Plk1 is not required for mitotic exit in these experiments. Although APC3 was also rapidly dephosphorylated in Plk1-depleted cells, Aurora A was not degraded (Fig. 5A). Importantly, the dephosphorylation of Cdh1 that occurs during mitotic exit, was dependent on Plk1 (Fig. 5A). The slower migrating Cdh1 band did represent hyperphosphorylation, since phosphatase treatment did result in an efficient downshift (Fig. 5C).

To investigate the function of hCdc14A *in vivo*, we utilized RNAi hairpins that efficiently down-regulated ectopic hCdc14A expression (Fig. 5B). When hCdc14A-depleted cells were assayed for APC/C-Cdh1 activity using live cell imaging, we observed a significant delay in the degradation of Aurora A-YFP A (Fig. 5B), indicating that hCdc14A plays a role in efficient activation of APC/C-Cdh1 *in vivo*.

We next assessed whether Plk1 controls Cdh1 through regulation of hCdc14A. To test the possibility that Plk1 controls hCdc14A directly, we investigated whether hCdc14A interacts with Plk1. Using GFP immunoprecipitations to pull down GFP-hCdc14A, we could co-immunoprecipitate endogenous Plk1 (Fig. 5D). The interaction between Plk1 and its binding partners was shown to be phosphorylation-dependent which often requires priming by Cyclin-dependent kinases [50], [51]. We therefore examined whether the interaction between Plk1 and hCdc14A required phosphorylation of S245, a conserved Cdk consensus phosphorylation site (Fig. 5E). Mutation of the S245 phosphorylation site did not disturb the interaction between Plk1 and hCdc14A, indicating that other kinases or other phosphosites may direct the binding between Plk1 and hCdc14A, or that other hCdc14A-associating proteins provide a docking site for Plk1. When we incubated recombinant hCdc14A with Plk1, Cdc14A was efficiently phosphorylated by Plk1 *in vitro* (Fig. 5F). This finding indicates that hCdc14A might be directly regulated by Plk1 (Fig. 5F, also see Suppl. Fig. S1F, G). Yuan *et al*. reported that S351 and S363 within human hCdc14A were phosphorylated after Plk1 addition [52]. Importantly, immuno-precipitated hCdc14A S351A–S363A was no longer phosphorylated by recombinant Plk1 (Fig. 5F). We next set out to study the functional relevance of Plk1-mediated phosphorylation of hCdc14A for APC/C-Cdh1 activation. To this end, both non-phosphorylatable or phospho-mimicking mutants of hCdc14A were expressed in Plk1-depleted U2OS cells and endogenous Aurora A levels were analyzed by FACS (Fig. 5G, also see Suppl. Fig. S2E). Expression of hCdc14A or the phospho-mutants only resulted in a minor decrease in Aurora A levels in mitotic cells, indicating that these mutants do not significantly inhibit accumulation of Aurora A in mitotic cells (Suppl. Fig. S2B, B). In addition, Cdc14A phosphorylation mutants localized to centrosomes as did wt-Cdc14A (Suppl. Fig. S2A and data not shown). Mitotic cells were subsequently collected by gentle shake-off and forced to exit mitosis by Roscovitine treatment (Fig. 5G, also see Suppl. Fig. S2E). Again, Plk1-depleted cells were unable to efficiently degrade Aurora A. Importantly, the phospho-mimicking S351-363D hCdc14A mutant rescued the defect in Plk1-depleted cells to degrade Aurora A, indicating that the APC/C-Cdh1 is significantly more active in these cells (Fig. 5G, also see Suppl. Fig. S2E). In contrast, expression of the non-phosphorylatable S351-363A hCdc14A mutant failed to promote efficient Aurora A degradation in Plk1-depleted cells (Fig. 5G). In conclusion, our results reveal that Plk1 can control the degradation of Aurora A by the APC/C-Cdh1 through phosphorylation of hCdc14A.

## Discussion

Of the diverse mitotic functions of Plk1, its role in APC/C-activation is one of the least understood. However, several lines of evidence point towards a link between Polo-like kinases and the APC/C. Most notably, in budding yeast the Cdc5 polo-like kinase controls the release of Cdc14 from the nucleolus, and the subsequent activation of APC/C-Cdh1 [49]. In higher organisms, the situation is less clear. *In vitro* phosphorylation studies have shown that Plk1 can modify the APC/C [34], [35]. Furthermore, Plk1 is involved in the degradation of the APC/C-inhibitor Emi1 early in mitosis and through this mechanism Plk1 could indirectly control APC/C-mediated degradation [11], [13]. Studies of Plk1-depletion or inhibition revealed that Plk1 involvement in the activation of the spindle checkpoint-independent APC/C-Cdc20 is limited since degradation of Cyclin A during prometaphase is not compromised [35], [38], [43]. Previous studies addressing the activation of the spindle checkpoint-dependent APC/C-Cdc20, required for the degradation of Cyclin B1 and Securin, have also failed to show a requirement for Plk1 [35], [37], [38]. Here, we analyzed Cyclin B1 degradation biochemically as well as in live cells, as a measure of spindle-checkpoint-dependent APC/C-Cdc20 activity and report that Plk1 is not required for timely Cyclin B1 degradation. This is in apparent contradiction with earlier results from *Xenopus* extracts, where depletion the Plk1-homologue Plx1, resulted in inhibition of Cyclin B1 degradation [53]. However, these latter results represent APC/C regulation during the metaphase to anaphase transitions of meiosis-II, which might be regulated differentially [54]. In addition, we investigated a role for Plk1 in APC/C-Cdc20 activation under conditions where the spindle assembly checkpoint is never established or was inactivated.

Activity of the checkpoint-dependent APC/C-Cdc20 is followed by activation of APC/C-Cdh1 upon inactivation of Cyclin B1-Cdk1. Our data show that Plk1 is critically required for efficient degradation of the APC/C-Cdh1 target Aurora A. We show that Plk1 directly modulates Cdc14A, a known regulator of the APC/C-Cdh1. In addition, we show that degradation of Cdc20 is also dependent on Plk1, suggesting that Plk1 controls activation of the APC/C-Cdh1. However, we do not exclude the possibility that Plk1 directly regulates Aurora A. In such a scenario, direct modification of Aurora A by Plk1, could make Aurora A susceptible for degradation by the APC/C. Indeed, Plk1 has been shown to directly interact with Aurora A, albeit as a substrate rather than an upstream kinase [5].

The observed impaired APC/C-Cdh1 activation correlated with a failure to dephosphorylate Cdh1, which is thought to be dependent on a Cdc14-phosphatase. Indeed, we found that the failure in APC/C-Cdh1 activation is reverted by expression of a mutant of hCdc14A of which two Plk1 phosphorylation sites are converted into phospho-mimicking residues. This indicates that hCdc14A could be an important intermediate in Plk1-dependent activation of APC/C-Cdh1 and adds a novel Plk1-dependent path in the control over APC/C activity. Besides direct phosphorylation of several APC/C subunits and degradation of an APC/C inhibitor, our strongly suggest that Plk1 also controls the dephosphorylation and activation of the APC/C auxiliary protein Cdh1. Although both chemical inhibition (BI2536) as well as Plk1 RNAi both resulted in impaired Aurora A destruction, Plk1-depletion had a more dramatic effect on Aurora A degradation when compared to Plk1 catalytical inhibition or hCdc14A-depletion. Similarly, expressing the phospho-mutants of hCdc14A never fully reverted the defect in Aurora A degradation, which suggests that additional Plk1-dependent pathways might converge on APC/C-Cdh1, next to Cdc14A regulation. Interestingly, two mammalian homologues of budding yeast Cdc14, hCdc14A and hCdc14B have been described [55]. hCdc14A localizes to centrosomes, whereas hCdc14B localizes in the nucleolus [55]–[58]. Until recently, only hCdc14A was tested for its ability to dephosphorylate and activate Cdh1 [47]. However, the Pagano lab recently showed that hCdc14B is implicated in activating APC/C^Cdh1^ in G2-phase in response to DNA damage [59]. It would therefore be interesting to see whether hCdc14B could also have a role in the activation of APC/C^Cdh1^ in mitosis, together with hCdc14A. In addition to Cdc14A, Plk1 has been described to regulate the APC/C-Cdh1 inhibitor Emi1 [11]–[13]. Since Plk1 controls Emi1 degradation at the onset of mitosis, we cannot exclude that Emi1 also contributes to Aurora A stabilization in cells lacking Plk1 activity. Addressing the independent roles of Emi1 and Cdc14 in APC/C-Cdh1 activation remains difficult as these pathways are intertwined [59] and analysis of the role of Emi1 in this process is experimentally difficult as Emi1 depletion precludes mitotic entry (data not shown).

It is interesting to note that Plk1 inhibition with a small molecule inhibitor had a weaker effect on Aurora A stability, compared to depletion of Plk1, hinting towards a structural role in complex formation between Cdh1 and its phosphatase. This could also explain the incomplete reversion of Plk1 depletion by a phospho-mimicking hCdc14A. Indeed, Plk1 could interact with Cdh1 (data not shown). However, we have not yet been able to address whether Plk1 can bind hCdc14A and Cdh1 at the same time or whether they bind in a mutually exclusive fashion.

The mechanism of APC/C-Cdh1 activation in mammalian cells that we describe here has similarities to the pathway promoting mitotic exit in budding yeast. In yeast, Cdh1 also requires removal of Cdk1-phosphorylation to activate the APC/C, and this dephosphorylation is mediated by the yeast Cdc14 phosphatase [26], [33], [48]. The budding yeast polo-like kinase Cdc5 promotes Cdc14 release from the nucleolus through regulation of the MEN and FEAR networks [60]–[65] and once released from the nucleolus, Cdc14 dephosphorylates Cdh1, so it can activate the APC/C [25], [26], [33], [48]. Our results indicate that control of Cdh1 by Plk1 through Cdc14A may highlight aspects of an evolutionary conserved part of APC/C regulation. However, clearly not all aspects of APC/C regulation are conserved. Activation of the budding yeast Cdc14-Cdh1 pathway requires a specific spindle orientation, in which one of the spindle poles is located in the daughter bud [49]. Such spatial regulation of mitotic exit by the MEN network appears unique to budding yeast [49]. Also, activation of Cdc14 requires release from its inhibitor Cfi1/Net1, which occurs in the nucleolus, a structure that is not present during a mammalian mitosis [66], [67]. Since hCdc14A localizes to the centrosome and no human homolog of Cfi1/Net1 has been identified, the direct inhibitory mechanisms for hCdc14A is unknown. In budding yeast, however, two signaling networks are involved in release of Cdc14 from the nucleolus; the MEN (mitotic exit network) and the FEAR (Cdc fourteen early anaphase release) network [64], [66], [67]. The budding yeast MEN network is under the control of spindle positioning in a manner that is not present in mammalian cells, but the human genome does harbor homologues of both the FEAR network (Plk1, separase) and the MEN network (GAPCenA, WARTS/LATS1, Mob1A and centriolin). The function of these homologues in mitotic exit is largely unexplored, but it is surprising that all these proteins localize to centrosomes [68]–[72]. Perhaps the centrosome has adapted certain functions from the nucleolus during evolution.

Finally, our results point towards feedback mechanisms in which Plk1 supports the inactivation of its two activating proteins Bora and Aurora A by initiating their proteasomal destruction through different pathways. Because Plk1 is only partially destabilized after mitosis, this may provide a mechanism to robustly prevent re-activation of Plk1 in G1 and S phase. It also would be interesting to see whether feedback mechanisms between Plk1 and APC/C-Cdh1 regulate Aurora A stability when cells are recovering after DNA damage in G2 phase, to re-initiate the cell cycle [5]. These interesting feedback mechanisms require further studies.

## Methods

### Cell culture, transfection and synchronization

Human osteosarcoma (U2OS) cells were cultured in DMEM-Glutamax (Gibco), complemented with 6% FCS, Penicillin and Streptomycin. Cells were transfected for 18 h using a standard calcium-phosphate protocol. Where indicated cells were blocked at the G1-S transition by incubation in thymidine (2.5 mM, Sigma) for 24 h. In order to allow synchronized progression through the cell cycle, thymidine was extensively washed away and replaced with pre-warmed medium. The Eg5 inhibitors Monastrol (0.2 mM, Sigma) and S-Trityl-L-Cysteine (STLC, 20 µM, NovaBiochem) were used to prevent centrosome separation. SP600125 (Biomol, 10 µM) was used to inhibit Mps1 and override the mitotic checkpoint, Roscovitine (Biomol, 50 µM) and BI2536 (kind gift from Boehringer Ingelheim, 100 nM) was used to inhibit Plk1.

### RNAi, Plasmids and antibodies

pSuper vectors producing siRNA's against Plk1, Mad2 and BubR1 were constructed and described previously [38]. On-target plus RNAi pools of 4 independent oligo's directed against human Plk1 were from Dharmacon. pS-hCdc14A was designed to target TCTCACCATTCTCGACTGT. Aurora A-YFP was kindly provided by Dr. C. Lindon (University of Cambridge, United Kingdom) and described previously [29]. Myc-hCdc14A was kindly provided by Dr. J. Lukas (Danish Cancer Society, Denmark) and described previously [58], GFP-Cyclin B1-NT and GFP-Cyclin B1 NT DM were kindly provided by Dr. M. Brandeis (the Hebrew University of Jerusalem, Israel) and described previously [39]. Cyclin B1-Cherry was made by ligating a Cyclin B1 fragment from a Cyclin B1-Venus construct, described in [21], into a Cherry-containing variant originating from the Clontech N1 vector. Use of Spectrin-GFP and pBabePuro were described previously [38]. Plasmids expressing GST-hCdc14A and Rabbit anti-hCdc14A antibody were kindly provided by Dr. U. Gruneberg (Max-Planck-Institute, Martinsried, Germany) and published previously [73]. GFP-hCdc14A was kindly provided by dr. J. Dixon and described previously [73]. GFP-hCdc14A-S351-363A, GFP-hCdc14A-S351-363D and GFP-Cdc14A-S254A were made using site-directed mutagenesis and validated by automated DNA sequencing. Mouse anti-MPM-2, and rabbit anti-Plk1 were from Upstate Biotechnology Inc. (Lake Placid, NY). Rabbit anti-gamma-Tubulin mouse anti-Cyclin B1, rabbit anti-Cdc20 and rabbit anti-Cdk4 were from Santa Cruz Biotechnology (Santa Cruz, CA). Rabbit anti-Aurora A was from Cell Signaling Inc, (Beverly, MA). Cy5-conjugated anti-mouse antibodies were from Jackson Immunoresearch Laboratories (Westgrove, PA). Rabbit anti-APC3 was from Becton Dickinson Transduction labs. Mouse anti-Cdh1 was from Neomarkers. Mouse anti-beta-Actin and rabbit anti-GFP was from Roche. Anti-Mouse Alexa-647 and Anti-Rabbit Alexa-488 were from Molecular Probes. Rabbit anti-APC2 was a kind gift from Hongtao Yu (University of Texas, Southwestern Medical Center, Dallas, US).

### Analysis of protein degradation

Biochemical analysis of transfected cells was performed as follows; U2OS cells were transfected with 10 µg of the indicated pSuper vectors in combination with 1 µg of the puromycin resistance marker pBabePuro. 18 h after transfection, cells were incubated for 24 h in thymidine (2.5 mM) and puromycin (2 µg/ml) to select for transfected cells. Thymidine and puromycin were washed away thoroughly, and at indicated time points cell lysates were obtained for kinase assays and western blotting. Alternatively, protein degradation was studied in mitotic cells (obtained by mitotic shake off after 16 h nocodazole treatment), which were treated with 50 µM Roscovitine for 2 h to allow mitotic exit. In order to analyze live protein degradation, U2OS cells were cultured on Wilco Wells (Wilco Well, Amsterdam, the Netherlands) and transfected with 1 µg of the indicated pSuper constructs in combination with 0.1 µg Aurora A-YFP or 0.1 µg of indicated Cyclin B1 constructs. 18 h after transfection, cells were blocked in thymidine for 24 h. At 10 h after release from thymidine block, cells were transferred to the heated culture chamber (37°C, 5% CO_2_) of a Zeiss Axiovert 200 M microscope equipped with a 0.55 numerical aperture (N.A) condenser and a 40× Plan-Neo DIC objective (N.A. = 1.3). Using Metamorph software (Universal Imaging, Downington, PA), 12 bits DIC images (100 msec exposures to halogen light) yellow, green or red fluorescence (100 msec exposures to blue light) were captured with a Photometrics Coolsnap HQ charged-coupled device (CCD) camera set at gain 1.0 (Scientific, Tucson, AZ) with appropriate filter cubes (Chroma Technology Corp.) to select specific fluorescence. Quantitative analysis of fluorescent Cyclin B1 and Aurora A-YFP was done using Metamorph software. In short, after background subtraction, integrated fluorescence levels were determined of the total area of individual cells and referenced at indicated cell cycle phases.

### Phosphatase assay

U2OS cells were transfected with 10 µg pS-Plk1. 60 hrs after transfection, cells that were arrested in mitosis were isolated by mitotic shake-off and lysed in E1A Lysis Buffer without phosphatase inhibitors. Extracts were split and incubated at 30 degrees Celsius for 40 minutes in the presence or absence of 15 units of lambda-phosphatase (Upstate Biotechnology Inc., Lake Placid, NY) after which the reaction was stopped by adding SDS-sample buffer.

### Flow cytometry

Cells were transfected with 10 µg of indicated pSuper constructs in combination with 1 µg of Spectrin-GFP or with 1 µg of GFP-hCdc14A. At indicated time points after release from thymidine block or after Roscovitine addition, cells were fixed in ice-cold 70% ethanol. After washing away ethanol, cells were stained with anti-MPM-2 or anti-Aurora A and counterstained with Alexa-647 or Alexa-488 -conjugated anti-mouse anti-rabbit antibodies. DNA was stained through Propidium/RNAse treatment. For each time point 10^4^ events were analyzed on a Becton Dickinson FACScalibur using cell quest software. Data were analyzed using MoFlow software.

### Kinase assays

Cyclin B1-associated kinase assays were performed as described in [74]. In brief, U2OS cells were harvested and lysed in E1A-lysis buffer. Subsequently, Cyclin B1-Cdk1 was immunoprecipitated from 5 µg of total lysate and kinase activity towards Histone H1 was measured using [gamma-^32^P] ATP. For Plk1 kinase assays, 2 ng recombinant human Plk1 (aa 36–346) was incubated with GST-hCdc14A or with GFP-Cdc14 IPs (Fig 5). Kinase activity was measured using [gamma-^32^P] ATP.

## Supporting Information

Figure S1 A–C U2OS cells were transfected with 1 µg of either GFP-Cyclin B1-NT or GFP-Cyclin B1-NT-DM. At indicated time points, fluorescence light and DIC images were captured. B, D Fluorescence levels from Fig. S1A/C were quantified using Metamorph software. Fluorescence levels at metaphase were arbitrarily set at 100% and shown standard error is based on three independent experiments. E. U2OS cells were transiently transfected with 1 µg Aurora A-YFP and 10 µg pS-Mad2. 18 h after transfection, cells were incubated for 24 h in thymidine. 10 h after washing away thymidine, cells were transferred to the heated stage of a time-lapse microscope. At indicated time points, DIC and fluorescent images were recorded. F. GST-hCdc14A was produced in DH5α cells and purified on Gluthation beads. Washed and eluted GST-hCdc14A is analyzed in SDS-PAGE. G. GST-Cdc14A was incubated with recombinant His-Plk1 T210D and analyzed by autoradiography. Arrowsheads indicate Plk1 autophosphorylation and hCdc14A phosphorylation.
(10.21 MB TIF)

Figure S2 A. U2OS cells were transiently transfected with 1 µg of indicated GFP-Cdc14A phosphorylation mutants. 48 h after transfection, cells were fixed and stained for gamma-tubulin. Representative images of interphase and mitotic cells are shown. B. U2OS cells were transfected with pS-Plk1 in combination with GFP-wt-Cdc14A, S351,363A Cdc14A or S351,363D Cdc14A. 36 h after transfection, nocodazole was added to cell cultures. After 16 h, mitotic cells were collected by shake-off. Mitotic cells were fixed in ethanol and stained with anti-Aurora A-Alexa-647. Number of Aurora A-positive cells is plotted (the mean and SEM of 3 experiments are plotted). C. Cells were treated with pS-Plk1 and processed as in Figure 4B, but complemented with 0.2 µg WT non-targetable Myc-Plk1 (WT-Plk1). Western blotting was conducted using indicated antobodies. D. U2OS cells were transfected with 1 µg of Aurora A-YFP A, 0.1 µg of Cyclin B1-Cherry and 0.1 µg of pS-Mad2. Cells were released from a thymidine for 16 hours, and at indicated time-points fluorescence images were obtained. Arrowheads indicate mitotic entry and anaphase onset. E. U2OS cells were transfected with pS-Plk1 in combination with GFP-Cdc14A S351,363A or GFP-hCdc14A S351,363D. 18 h after release from a thymidine block, mitotic cells were collected and replated in medium containing Roscovitine for 4 hours. Cell lysates were analyzed for Aurora A, Plk1, GFP and Cdk4 by Western blotting.
(10.26 MB TIF)
