# Supplementary figures and images for: Polo-Like Kinase-1 Controls Aurora A Destruction by Activating APC/C-Cdh1
Source: PLoS One. 2009 Apr 23;4(4):e5282. doi: 10.1371/journal.pone.0005282 (PMC2668763; doi:10.1371/journal.pone.0005282)

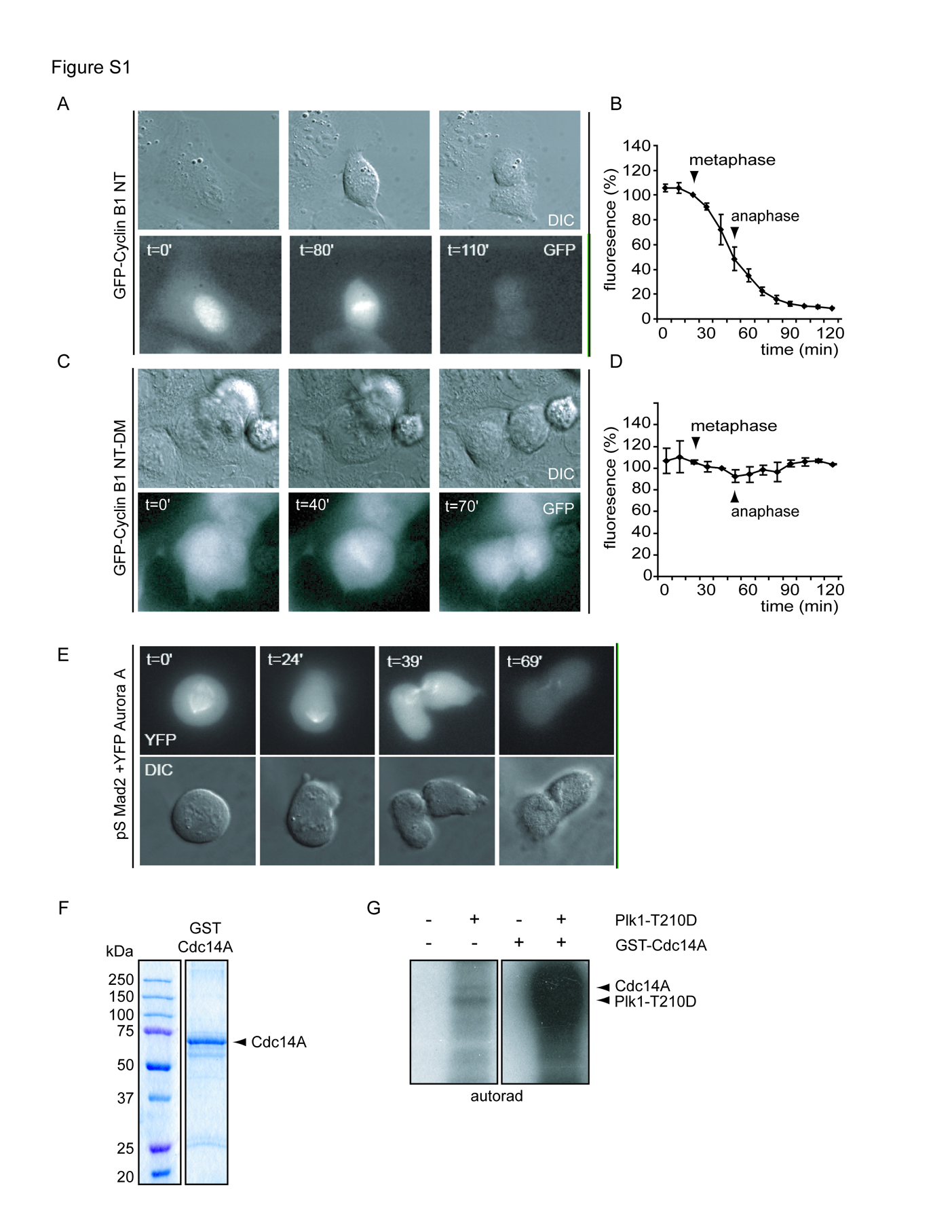

Supplement: Figure S1 — A–C U2OS cells were transfected with 1 µg of either GFP-Cyclin B1-NT or GFP-Cyclin B1-NT-DM. At indicated time points, fluorescence light and DIC images were captured. B, D Fluorescence levels from Fig. S1A/C were quantified using Metamorph software. Fluorescence levels at metaphase were arbitrarily set at 100% and shown standard error is based on three independent experiments. E. U2OS cells were transiently transfected with 1 µg Aurora A-YFP and 10 µg pS-Mad2. 18 h after transfection, cells were incubated for 24 h in thymidine. 10 h after washing away thymidine, cells were transferred to the heated stage of a time-lapse microscope. At indicated time points, DIC and fluorescent images were recorded. F. GST-hCdc14A was produced in DH5α cells and purified on Gluthation beads. Washed and eluted GST-hCdc14A is analyzed in SDS-PAGE. G. GST-Cdc14A was incubated with recombinant His-Plk1 T210D and analyzed by autoradiography. Arrowsheads indicate Plk1 autophosphorylation and hCdc14A phosphorylation. (10.21 MB TIF) [file pone.0005282.s001.tif]

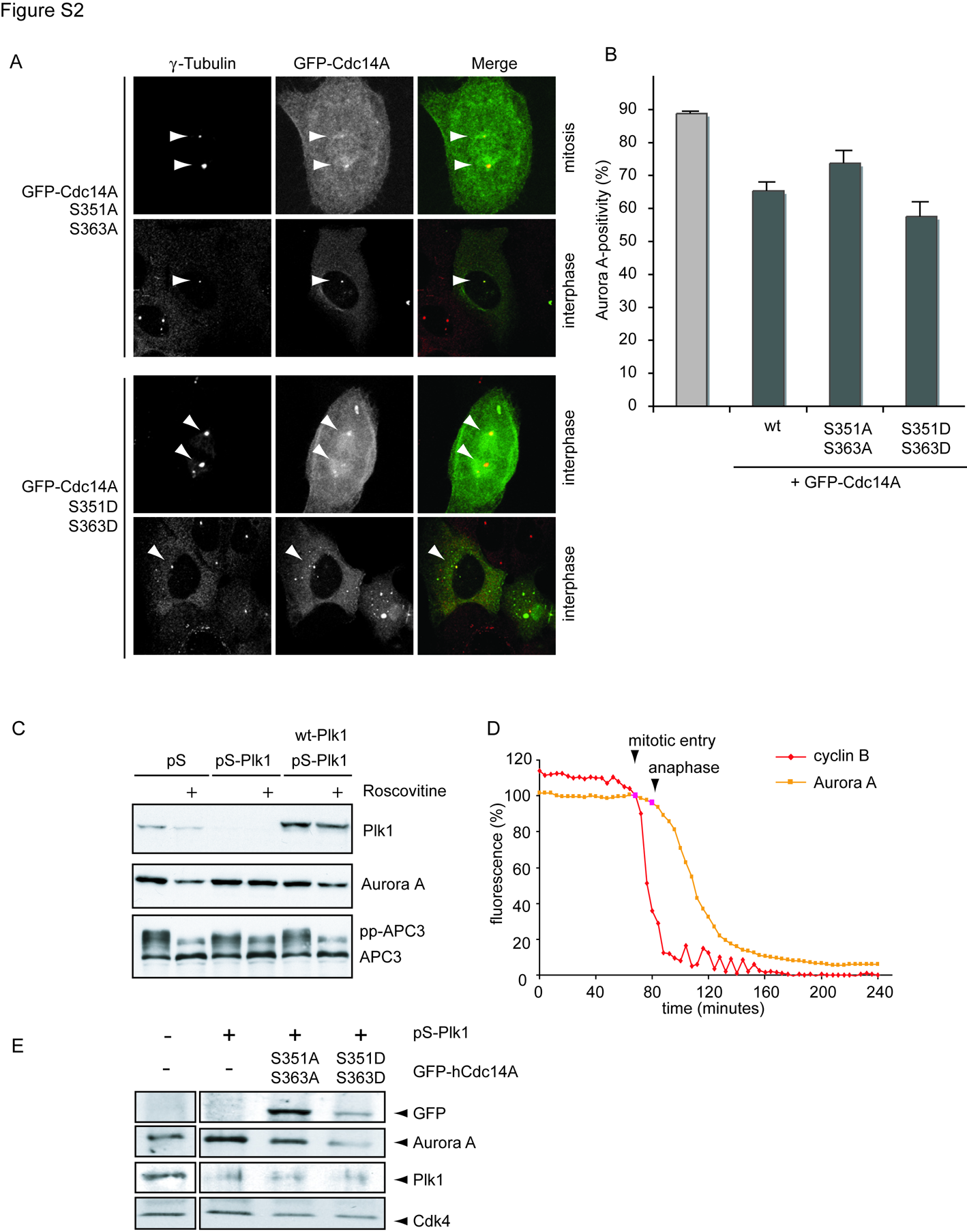

Supplement: Figure S2 — A. U2OS cells were transiently transfected with 1 µg of indicated GFP-Cdc14A phosphorylation mutants. 48 h after transfection, cells were fixed and stained for gamma-tubulin. Representative images of interphase and mitotic cells are shown. B. U2OS cells were transfected with pS-Plk1 in combination with GFP-wt-Cdc14A, S351,363A Cdc14A or S351,363D Cdc14A. 36 h after transfection, nocodazole was added to cell cultures. After 16 h, mitotic cells were collected by shake-off. Mitotic cells were fixed in ethanol and stained with anti-Aurora A-Alexa-647. Number of Aurora A-positive cells is plotted (the mean and SEM of 3 experiments are plotted). C. Cells were treated with pS-Plk1 and processed as in Figure 4B, but complemented with 0.2 µg WT non-targetable Myc-Plk1 (WT-Plk1). Western blotting was conducted using indicated antobodies. D. U2OS cells were transfected with 1 µg of Aurora A-YFP A, 0.1 µg of Cyclin B1-Cherry and 0.1 µg of pS-Mad2. Cells were released from a thymidine for 16 hours, and at indicated time-points fluorescence images were obtained. Arrowheads indicate mitotic entry and anaphase onset. E. U2OS cells were transfected with pS-Plk1 in combination with GFP-Cdc14A S351,363A or GFP-hCdc14A S351,363D. 18 h after release from a thymidine block, mitotic cells were collected and replated in medium containing Roscovitine for 4 hours. Cell lysates were analyzed for Aurora A, Plk1, GFP and Cdk4 by Western blotting. (10.26 MB TIF) [file pone.0005282.s002.tif]
